# Associations between classical Alzheimer's Disease biomarkers and key proteins defining potential pathophysiological mechanisms – A multi‐analyte proteomic analysis

**DOI:** 10.1002/alz70856_106823

**Published:** 2026-01-08

**Authors:** Joshua L Gills, Hubert Leo, Omonigho M Bubu, The ARIC Neurocognitive Biomarker Workgroup

**Affiliations:** ^1^ NYU Grossman School of Medicine, New York, NY, USA; ^2^ New York University Langone Health, New York City, NY, USA

## Abstract

**Background:**

Plasma biomarkers like amyloid‐beta Aβ42/Aβ40, *p*‐tau isoforms, and NfL are promising for assessing AD risk and pathology. Emerging platforms such as Nucleic Acid‐Linked Immuno‐Sandwich Assay (NULISA) enable precise detection of low‐abundance proteins, offering insights into AD mechanisms. We examined associations between Alamar's NULISA proteins related to AD (amyloid, tau, neurodegeneration, and inflammation [ATN & I]) and Alamar NULISA's 126 CNS panel proteins to evaluate internal correlations.

**Method:**

We conducted a cross‐sectional analysis of plasma samples from 116 ARIC participants (part of the ARIC calibration panel). Biomarkers were quantified using the ALAMAR NULISA platform, measuring 126 CNS specific analytes. ATN & I biomarkers included: A (Aβ proteinopathy) – Aβ‐42, Aβ‐40, and Aβ‐42/40; T (phosphorylated and secreted AD tau) ‐ *p*‐tau217, *p*‐tau181, *p*‐tau231 and their ratios with Aβ‐42; **N** (injury, dysfunction, or degeneration of neuropil) – NfL and Total Tau; and **I** (inflammation ‐ Astrocytic activation) – GFAP. Multiple linear regression was used to examine ATN & I biomarker associations with the 126 CNS NULISA proteins. Bonferroni correction was used to adjust for multiple comparisons. Analyses adjusted for age, sex, APOE ε4 status, education, and glomerular filtration rate (GFR). Spearman's Rho correlations were utilized to examine ATN & I protein associations amongst themselves.

**Result:**

Of the 116 participants (age = 74.3 ± 4.7), 50% were female, and 88.8% were cognitively normal; 29.7% were APOE4 carriers. Aβ proteinopathy proteins showed moderate to strong correlations with each other (ρ = .52‐.88), mild to moderate correlations with phosphorylated and secreted AD tau markers (ρ = .20‐.68), and negative correlations with tau ratios with Aβ‐42 (‐.14 to ‐.55). The Aβ‐42/40 ratio showed mild correlations with other biomarkers. Among 126 Alamar proteins, 38 plasma proteins were associated with NfL, 37 with Aβ‐42 and Aβ‐40, and 35 with Tau. Top hits included IL‐33, MAPT, VEGFD, TAFA‐5, FOLR1, and GDB/1.

**Conclusion:**

AD‐specific proteins are moderately to strongly correlated with each other on the Alamar platform. The most significantly associated proteins with ATN & I biomarkers within the platform included markers subserving inflammation, connective tissue integrity, and vascular function. Follow up studies should be longitudinal with individuals across the AD continuum.

